# Emetine in Haemoptysis

**Published:** 1914-06

**Authors:** 


**Emetine in Haemoptysis.** C. Flandin, quoted in the Prescriber, Jan., 1914, influenced by the reports of cessation of intestinal haemorrhage in cases of dysentery and hepatic abscess treated by emetine, tried an injection of 4 centigrams in 1 c.c. of water, for haemoptysis, with good results and has continued this treatment in several cases. The haemoptysis is sometimes very promptly relieved. Usually even without recurrence of haemorrhage, the injection is repeated after 12 hours and daily for four doses. No untoward symptoms were noted.

